# The Impact of Methotrexate on Patients With Rheumatoid Arthritis-Associated Interstitial Lung Disease: A Systematic Literature Review

**DOI:** 10.7759/cureus.96328

**Published:** 2025-11-07

**Authors:** Jeffrey Shin, Michael Parker, Jordan J Ditchek, Marc M Kesselman

**Affiliations:** 1 Medicine, Dr. Kiran C. Patel College of Osteopathic Medicine, Nova Southeastern University, Davie, USA; 2 Pharmacology, Dr. Kiran C. Patel College of Allopathic Medicine, Nova Southeastern University, Davie, USA; 3 Medical Education, Dr. Kiran C. Patel College of Allopathic Medicine, Nova Southeastern University, Fort Lauderdale, USA; 4 Rheumatology, Dr. Kiran C. Patel College of Osteopathic Medicine, Nova Southeastern University, Davie, USA

**Keywords:** disease-modifying antirheumatic drugs, interstitial lung disease in rheumatoid arthritis, methotrexate, pulmonary fibrosis, rheumatoid arthritis, rheumatoid arthritis-associated interstitial lung disease, systematic review, treatment of rheumatoid arthritis

## Abstract

Rheumatoid arthritis (RA), a long-term autoimmune disorder, continues to affect millions worldwide and is often accompanied by interstitial lung disease (ILD). This serious complication can dramatically worsen patient outcomes. Methotrexate (MTX), one of the most prescribed treatments for RA, has been widely used for its effectiveness but criticized for its potential respiratory side effects. Questions remain about whether MTX contributes to the onset or progression of ILD in this population. To explore this issue, 19 studies published between 2010 and 2025 were reviewed, covering a total of 47,271 patients with RA who had received MTX. The included studies varied in design, ranging from observational and comparative cohort analyses to experimental animal models, and were drawn from diverse geographic populations, including several from East Asia.

According to the studies, five reported a link between MTX use and increased risk or worsening of RA-associated ILD (RA-ILD). The remaining 14 studies found no evidence of such an association, and some even suggested that MTX has protective properties reducing inflammation and limiting lung damage. Direct comparisons were complicated due to differences in study design, diagnostic criteria, patient demographics, and smoking status. Based on the literature collected from this review, most reports that have identified harmful effects of MTX were within the Asian population, hinting at a possible risk factor within the Asian population. The findings of these studies indicate that while concerns about MTX and ILD have some basis in literature, the collective findings fail to demonstrate a consistent or definitive risk. In fact, MTX may offer benefits for some patients when used appropriately. However, more well-controlled, multinational studies are needed to better understand how MTX interacts with lung tissue in RA and to determine which patients, if any, are at an elevated risk for developing ILD during treatment. This study aims to contribute to the evolving understanding of MTX on RA-ILD and highlights the importance of careful clinical judgment when initiating MTX therapy.

## Introduction and background

Rheumatoid arthritis (RA) is an autoimmune disease that has a significant global burden, with an estimated 17.6 million people living around the world with this diagnosis in 2020. However, with increased predispositions and environmental factors increasing susceptibility to diseases, the forecasted prevalence of RA is expected to reach 31.7 million individuals by 2050 [1]. RA is a systemic autoimmune disease that is characterized by synovial inflammation and hyperplasia, autoantibody production (rheumatoid factor and anti-citrullinated protein antibody (ACPA)), cartilage and bone destruction, and systemic features, including cardiovascular, pulmonary, psychological, and skeletal disorders [2]. Among the most common comorbidities of RA is interstitial lung disease (ILD), a condition that increases mortality risk in affected patients. RA-associated ILD (RA-ILD) is often progressive and frequently leads to disabling symptoms and respiratory failure, while methotrexate (MTX)-induced interstitial lung disease (MTX-ILD) is commonly an acute or subacute onset of symptoms such as coughing, dyspnea, and fever after starting methotrexate [3].

RA susceptibility is strongly linked to human leukocyte antigen (HLA) class 2 gene mutations, which encode proteins that present extracellular antigens to CD4+ T helper cells, which can influence the production of autoantibodies like ACPA [4]. Additionally, environmental factors such as smoking and silica dust are considered risk factors for the development of RA, both of which can interact with HLA alleles and lead to autoantibody production. Analysis of various stimuli that induce neutrophil activation and death revealed that perforin and the membrane attack complex (MAC), two immune-mediated membranolytic pathways, possess a unique capacity to reproduce patterns of hyper-citrullination observed in RA [5]. Citrullination is a normal reaction during apoptosis and can occur in all cells. However, a defect in the clearance system can occur due to massive cell death, resulting in the accumulation and leakage of citrullinated peptides. Citrullinated peptides can be recognized by the immune system to make anti-citrullinated protein antibodies (ACPAs) and temporarily activate the immunological tolerance of the lungs [6]. Additionally, the lung can become susceptible to autoimmune ILD by B-cell immunity after the loss of self-tolerance from the following events: (1) the inhalation of neoantigens, (2) generation of cross-reactive autoantibodies in a systemic disorder, or (3) expression of neoepitopes in airway or pulmonary tissue after bronchial or lung injury [7]. With respect to point 2, citrullinated peptides may also be produced in the lungs, causing an immune response [8]. A systematic review and meta-analysis showed that the existence of ACPA was significantly associated with RA-ILD incidence, and autoantibody levels were significantly increased in these patients [9]. The supposed mechanism by which this occurs is through the accumulation of citrullinated proteins in the bronchoalveolar lavage fluid of patients with RA-ILD, thereby causing an inflammatory response [9]. On the other hand, MTX-ILD often presents as a nonspecific interstitial pneumonia (NSIP) or bronchiolitis obliterans organizing pneumonia, and histopathology may show interstitial infiltrates with lymphocytes, histiocytes, eosinophils, and sometimes non-caseating granulomas. Management requires immediate cessation of methotrexate and initiation of glucocorticoids [3].

While treatment strategies for RA have been extensively studied, offering a variety of options and combinations, RA-ILD presents a unique challenge. Patients with RA-ILD are often excluded from randomized clinical trials, creating a critical gap in understanding practical treatment approaches for this population. Current treatments for RA span conventional synthetic disease-modifying anti-rheumatic drugs (csDMARDs), biologic DMARDs, and targeted synthetic DMARDs. These drugs ultimately interfere with critical pathways in the inflammatory cascade [10]. Methotrexate (MTX), for example, stimulates adenosine release from fibroblasts, rescues neutrophil adhesion, inhibits leukotriene B4 synthesis by neutrophils, inhibits local IL-1 production, reduces levels of IL-6 and IL-8, suppresses cell-mediated immunity, and inhibits synovial collagenase gene expression [10]. However, MTX’s utility in treating RA-ILD remains uncertain. Specifically, methotrexate is widely used in RA, and there are various recognized side effects, including lung toxicity [11]. The most associated lung injury described in association with MTX use is interstitial pneumonitis, which can lead to pulmonary fibrosis in patients who have been receiving long-term oral therapy for rheumatoid arthritis [12]. Although the exact mechanism of methotrexate in RA is unknown, it is thought to involve an increase in adenosine levels and signaling, leading to an intracellular cascade that promotes an overall anti-inflammatory state [13].

CsDMARDs are considered the most prescribed treatment for RA, with 77.2%-79.2% of patients being prescribed csDMARDs from 2016 to 2021. MTX is the most prescribed of the csDMARDs; however, questions regarding the effectiveness in treating and the potential exacerbation of RA-ILD by MTX are still raised to this day [14]. Growing evidence suggests that RA disease control is of paramount importance, as increased systemic disease activity is associated with increased mortality in patients with RA-ILD. Moreover, recent case reports have associated MTX with the prevalence and onset of RA-ILD, while clinical research has shown otherwise [15-19]. Although MTX-ILD and RA-ILD differ in the progression of their symptoms, this review investigates whether MTX exacerbates or prevents RA-ILD, highlighting the need for a deeper understanding and clarification of recent literature on the efficacy of MTX and its implications.

## Review

Methodology

Search Strategy

A systematic literature review was conducted on the recent literature between 2010 and 2025. The articles collected were sourced from the Biomedical Reference Collection: Comprehensive (EBSCOhost), CINAHL Complete (EBSCOhost), OVID, and Web of Science. The key search terms that were examined were “Rheumatoid arthritis” AND “Interstitial lung disease” AND “Methotrexate” OR “Conventional synthetic disease-modifying anti-rheumatic drugs.” Each article was examined through a two-step screening process. First, the title and abstract were evaluated to gauge the relevance to the topic of our searches. Next, we analyzed the full-text manuscript to extract the progression of RA-ILD for patients treated with methotrexate. The Nova Southeastern University library database was used to access various databases and articles.

Selection Criteria

**Figure 1:**
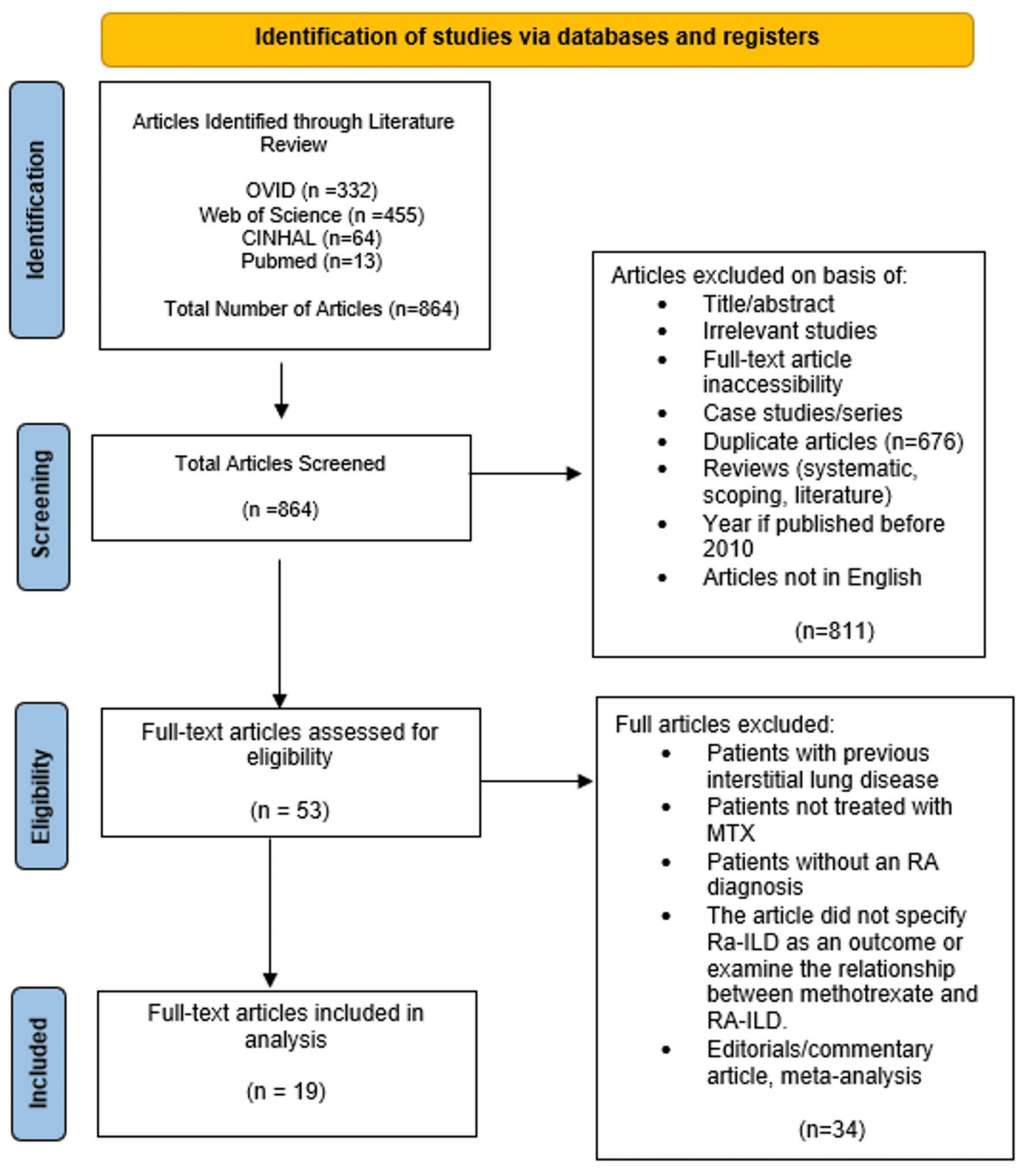
PRISMA indicating data selection PRISMA: Preferred Reporting Items for Systematic Reviews and Meta-Analyses, MTX: methotrexate, RA: rheumatoid arthritis, RA-ILD: rheumatoid arthritis-associated interstitial lung disease

The studies included in the review were case-control studies, randomized controlled trials, cross-sectional studies, animal studies, and cohort, prospective, and retrospective studies. The population included is rheumatoid arthritis patients being treated with methotrexate. The observed outcome was any diagnosis of RA-ILD following the treatment with methotrexate. Excluded studies were case studies, editorials, self-controlled case series, and systematic or scoping reviews. Excluded articles were those in which the patients had a pre-existing diagnosis of RA-ILD, if the patient had been previously treated with any treatment other than methotrexate, if the patient was treated in combination with methotrexate and any other rheumatic therapy, or if studies did not specify RA-ILD as an outcome. The Preferred Reporting Items for Systematic Reviews and Meta-Analyses (PRISMA) guidelines were followed and used to develop a diagram of selected articles (Figure 1) [20].

Data Extraction

Two authors independently screened all available studies for inclusion. For discrepancies, a third author was consulted to adjudicate and determine the result. Data from each study were extracted using a predefined data collection form, which included basic information (first author, year of publication, and study design) and patient characteristics (number of patients with RA, number of ILD outcomes, drug dosage and usage, age, sex, disease duration, RA disease activity, and follow-up time in the treatment and comparator groups).

Risk of bias for non-randomized studies was assessed using the ROBINS-I tool. For randomized studies, the Cochrane Risk of Bias Tool was employed. Any disagreements between reviewers were resolved through discussion.

Results

Within the 19 included studies, a total of 47,271 patients with RA receiving MTX were analyzed and examined for a causal relationship with RA-ILD. Of the 47,271 patients, a significant majority were female patients (71.6%). Of the 19 studies, 13 included comparator drugs of other csDMARDs (leflunomide and sulfasalazine) and other biologic DMARDs. Additionally, the types of ILD recognized were usual interstitial pneumonia (UIP) and nonspecific interstitial pneumonia (NSIP), with most studies using high-resolution computed tomography (HRCT)-confirmed ILD, although others possibly used International Classification of Diseases (ICD) coding or chest X-rays.

Outcome Analysis

A total of 19 studies were included in this systematic review, encompassing a range of retrospective and prospective cohort studies, case-control studies, cross-sectional analyses, and one animal model experiment. The sample sizes varied widely, from small single-center cohorts to large population-based registries involving tens of thousands of patients. The studies reported mixed results regarding the association between methotrexate use and RA-ILD.

Studies Reporting a Positive Association Between MTX and RA-ILD

**Figure 2:**
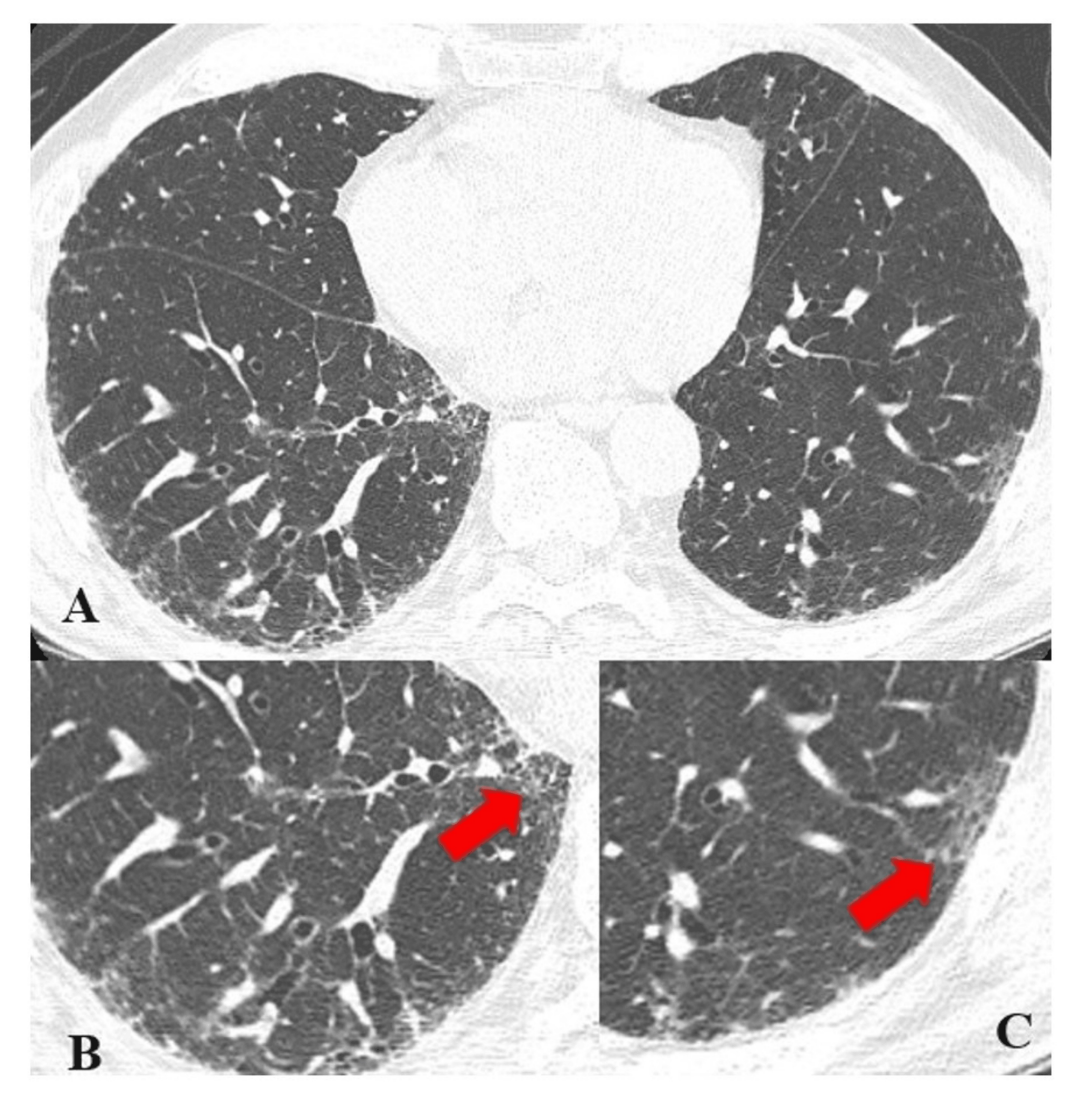
Mild ILD reticular opacities with peripheral/subpleural distribution (A) Axial CT image of the chest and magnified images of the periphery of the lung bases. (B and C) Small areas of reticular opacity (arrows) in this case, with bilateral subpleural distribution. ILD: interstitial lung disease, CT: computed tomography Image credit: Dr. Jordan Ditchek, MD (used with permission)

**Figure 3:**
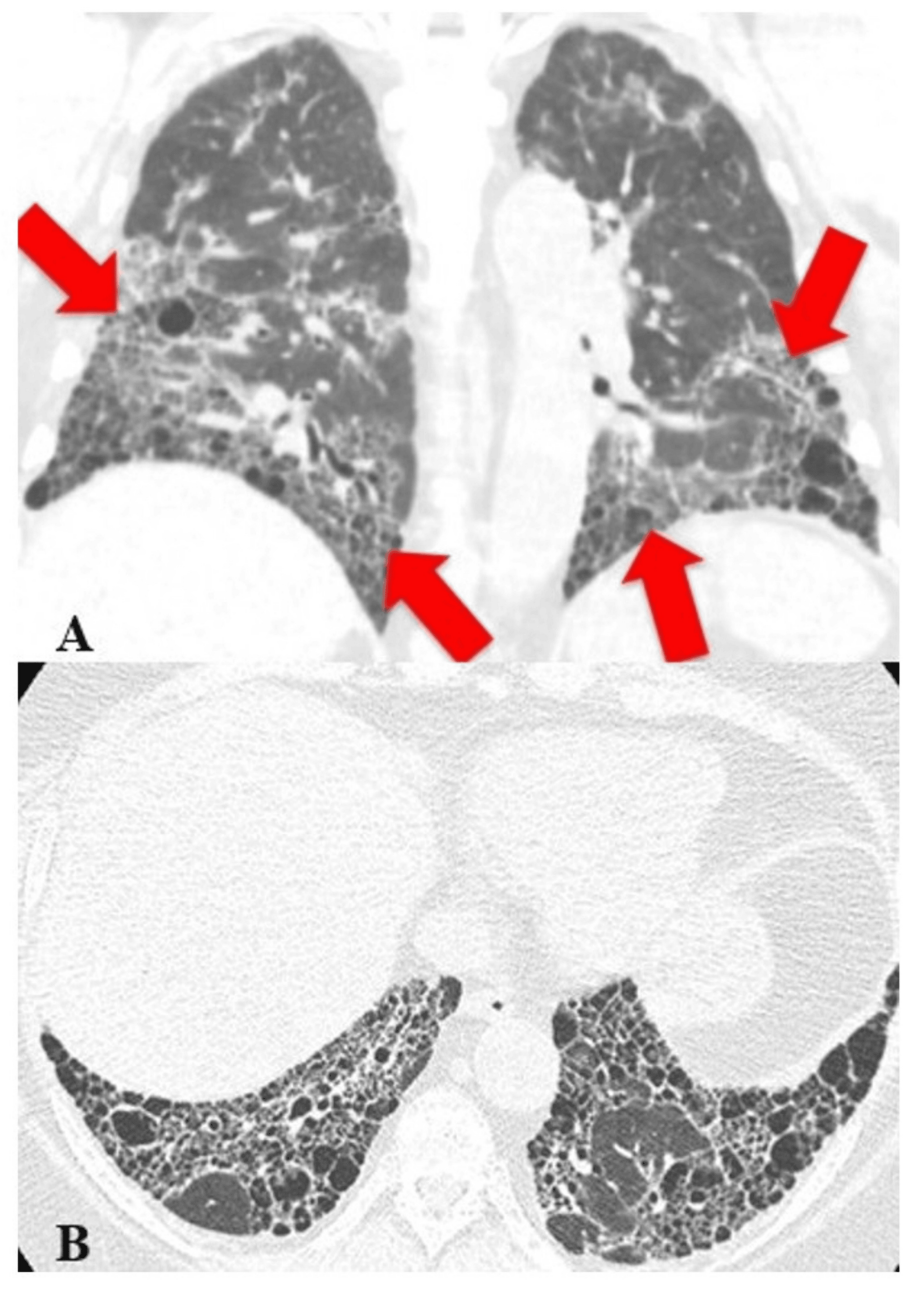
Moderate ILD with subpleural and basilar predominance (A) Axial CT image of the chest shows extensive confluent areas of reticular opacity in both lungs. (B) Coronal CT image highlights the bibasilar predominance of the involvement in this patient. ILD: interstitial lung disease, CT: computed tomography Image credit: Dr. Jordan Ditchek, MD (used with permission)

**Figure 4:**
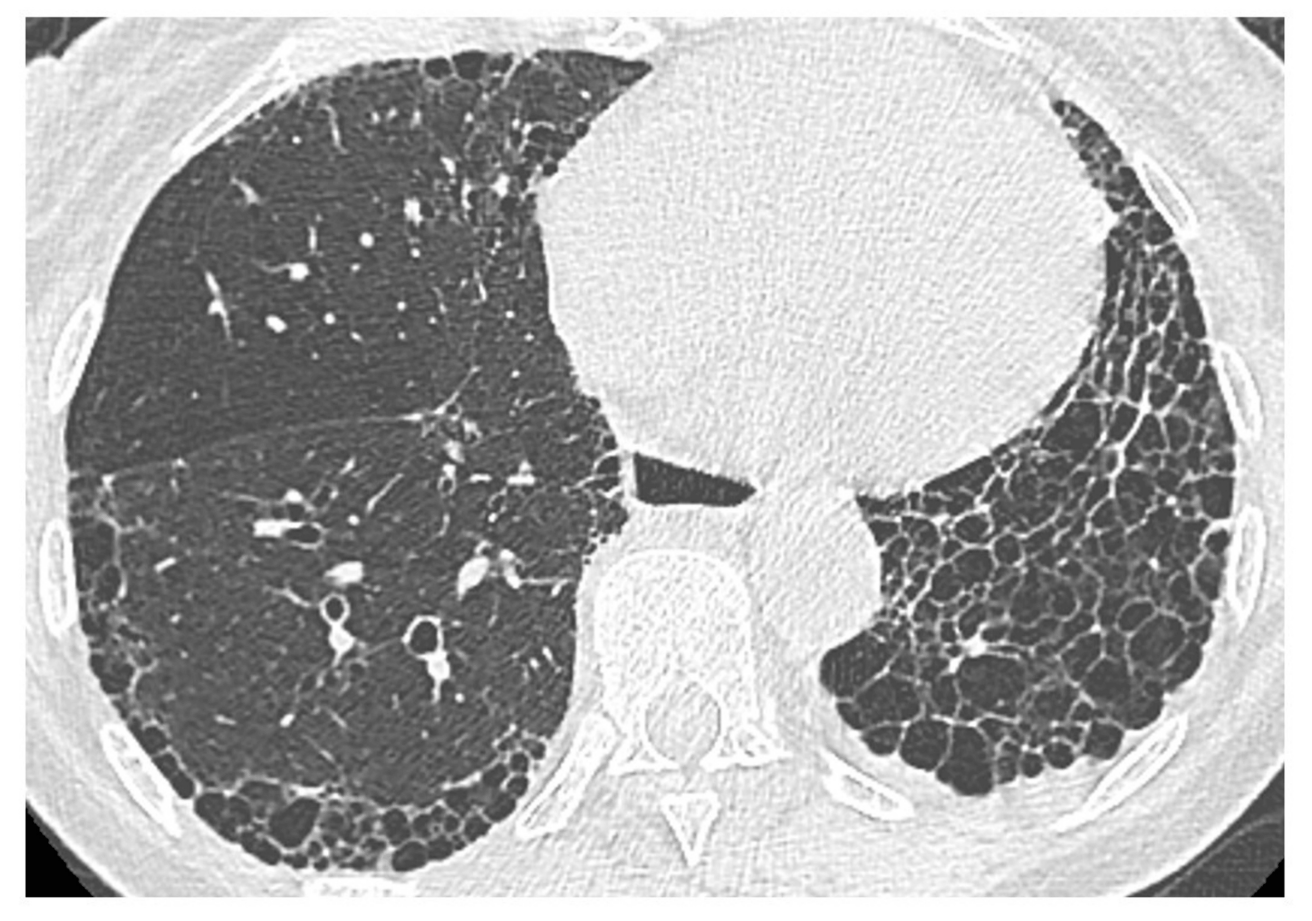
Advanced ILD with pulmonary fibrosis and honeycombing Axial CT image shows a “honeycomb” appearance posteriorly in the right lower lobe and throughout the visualized left lower lobe. Note the small, clustered cystic spaces with well-defined walls. ILD: interstitial lung disease, CT: computed tomography Image credit: Dr. Jordan Ditchek, MD (used with permission)

**Figure 5:**
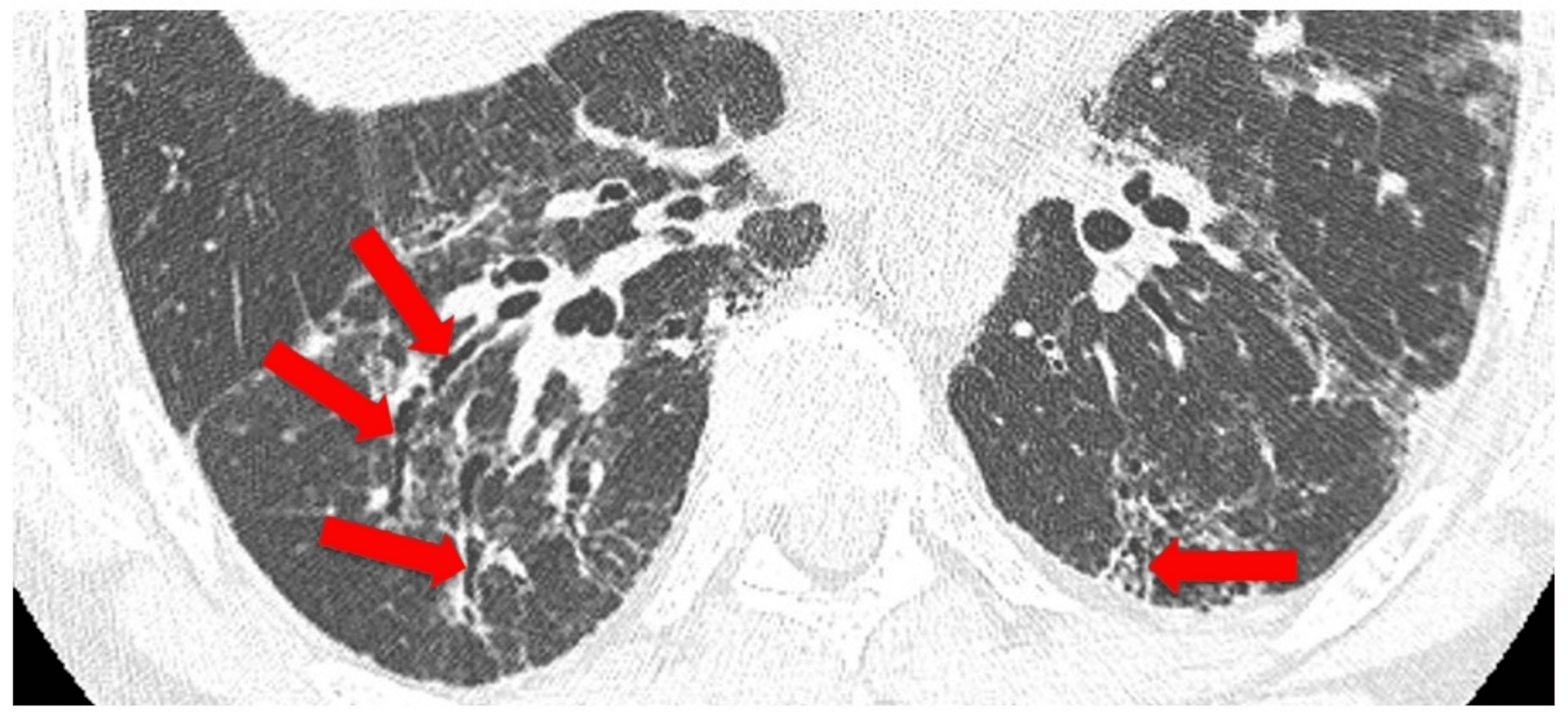
Advanced ILD with traction bronchiectasis Note the dilated and distorted bronchi (arrows), associated with the areas of reticular opacity in the lung parenchyma. ILD: interstitial lung disease Image credit: Dr. Jordan Ditchek, MD (used with permission)

Five studies reported findings suggestive of a potential association between MTX use and the development or exacerbation of RA-ILD (Table 1) [15-19]. For instance, Zhou et al. identified methotrexate as a statistically significant risk factor for RA-ILD development, with a positive standardized β coefficient in multivariate analysis, and 150 of 196 patients with RA-ILD were exposed to MTX [19]. Huang et al. similarly found that MTX use was associated with over twice the odds of lung disease (adjusted odds ratio (OR): 2.73, 95% confidence interval (CI): 1.37-5.61, p = 0.005) [17]. Hozumi et al. specifically linked MTX to an increased risk of acute exacerbation (AE) in RA-ILD (hazard ratio (HR): 3.04, 95% CI: 1.62-6.02, p = 0.001), noting that 55% of AE cases occurred in MTX users [18]. Figures 2-5 show axial CT images demonstrating the typical progression observed during significant symptom exacerbation. Moreover, Chang et al. used an SKG mouse model and single-cell transcriptomics to mechanistically demonstrate that MTX exacerbated pulmonary inflammation, fibrosis, and epithelial cell damage in a preclinical model of RA-ILD [15].

**Table 1:** Articles reporting a positive association between MTX and RA-ILD ACR/EULAR Criteria: American College of Rheumatology and the European League Against Rheumatism, csDMARDs: conventional synthetic disease-modifying anti-rheumatic drugs, bDMARDs: biological disease-modifying anti-rheumatic drugs, HRCT: high-resolution computed tomography, DLCO: diffusion lung capacity for carbon monoxide, UIP: usual interstitial pneumonia, TNFi: tumor necrosis factor inhibitors, CT: computed tomography, NSIP: nonspecific interstitial pneumonia, PFTs: pulmonary function tests, ATS/ERS: American Thoracic Society and the European Respiratory Society, MTX: methotrexate, RA-ILD: rheumatoid arthritis-associated interstitial lung disease, RF +: rheumatoid factor positive

Author and year (reference)	Number of patients receiving MTX	Comparator drug	Number of patients receiving comparator treatment	Study duration, weeks	Average age, years	Female sex, %	Number of ILD cases	Smoking history	RF +, %	Diagnosis modality	Type of ILD	Risk of bias	Study design
Chang et al. (2024) [15]	168	N/A	N/A	24	66.2	70.1	N/A	N/A	Chest CT and ACR/EULAR criteria	88.3	N/A	Moderate	Experimental animal study
Dinache et al. (2022) [16]	57	Other csDMARDs and bDMARDs	100	187	68.2	75.6	15	51	51	X-ray, HRCT, DLCO	UIP	Moderate	Retrospective, cross-sectional observational study
Huang et al. (2020) [17]	85	Prednisone, TNFi	71, 63	396	64.2	80	54	97	128	Chest CT	NSIP, UIP, subpleural fibrosis	High	Prospective, observational cohort study
Hozumi et al. (2013) [18]	10	Corticosteroids, immunosuppressants	29, 17	442	62	43	51	61	N/A	ACR, PFTs, HRCT	UIP, non-UIP	Moderate	Retrospective case-control study
Zhou et al. (2025) [19]	459	N/A	N/A	312	62	82.4	196	20	N/A	HRCT, ATS/ERS criteria	UIP	Moderate	Hospital-based retrospective cohort study

Studies Reporting No Association Between MTX and RA-ILD

Fourteen studies reported no statistically significant association between MTX use and the onset of RA-ILD (Table 2) [21-34]. For example, Chamizo-Carmona et al. [21], Juge et al. [26], Ibfelt et al. [25], and additional studies have found an inverse association, with MTX users being significantly less likely to develop RA-ILD across multiple cohorts (adjusted OR of 0.46 and 0.39 in discovery and replication cohorts, respectively) [22,23,24]. They found no association with AE risk (OR: 1.75, 95% CI: 0.76-4.00), while demonstrating a survival benefit for MTX users after AE (HR: 0.16, 95% CI: 0.04-0.72, p = 0.02). Robles-Pérez et al. [28] and Li et al. [27] observed that MTX was not associated with worsening diffusion capacity (DLCO) or increased risk of ILD in patients with early RA. Extensive cohort studies by Kiely et al. [29], Curtis et al. [31], and Sparks et al. [30] similarly found no increase in RA-ILD incidence or progression among MTX users. Notably, Izuka et al. [33] and Kur-Zalewska et al. [32] reported no difference in MTX exposure between patients with RA with and without ILD (60% versus 60.8%).

**Table 2:** Articles reporting no association between MTX and RA-ILD ACR/EULAR Criteria: American College of Rheumatology and the European League Against Rheumatism, csDMARDs: conventional synthetic disease-modifying anti-rheumatic drugs, bDMARDs: biological disease-modifying anti-rheumatic drugs, HRCT: high-resolution computed tomography, DLCO: diffusion lung capacity for carbon monoxide, UIP: usual interstitial pneumonia, TNFi: tumor necrosis factor inhibitors, CT: computed tomography, NSIP: nonspecific interstitial pneumonia, PFTs: pulmonary function tests, ATS/ERS: American Thoracic Society and the European Respiratory Society, ILD: interstitial lung disease, OP: organizing pneumonia, LIP: lymphoid interstitial pneumonia, FVC: forced vital capacity, CRF: chronic respiratory failure, CXR: chest X-ray, ICD-9: International Classification of Diseases, RA-ILD: rheumatoid arthritis-associated interstitial lung disease, IIP: idiopathic interstitial pneumonia, ILD-AE: interstitial lung disease acute exacerbation, HCQ: hydroxychloroquine, LEF: leflunomide, SSZ: sulfasalazine, MTX: methotrexate, RF +: rheumatoid factor positive

Author and year (reference)	Number of patients receiving MTX	Comparator drug	Number of patients receiving comparator treatment	Study duration, weeks	Average age, years	Female sex, %	Number of ILD cases	Smoking history	RF +, %	Diagnosis modality	Type of ILD	Risk of bias	Study design
Chamizo-Carmona et al. (2018) [20]	301	N/A	N/A	543.2	49.6	67.1	15	8	141.7 number	HRCT	UIP, NSIP	Moderate	Case-control study
Genc et al. (2023) [21]	41	Rituximab	4	52.1	59	73.56	5	4	57	HRCT, PFT, and DLCO	UIP, NSIP	Moderate	Retrospective, cross-sectional observational study
Severo et al. (2022) [22]	124	N/A	N/A	39	61	89.6	49	62	86.2	CT, PFTs, laboratory data	UIP	Serious	Retrospective, cross-sectional study
Kim et al. (2022) [23]	79	HCQ, LEF, SSZ	76, 62, 45	167	64	52.9	170	45.9	91.6	HRCT, spirometry, ACR/EULAR criteria	UIP, NSIP, OP	Moderate	Retrospective cohort study
Ibfelt et al. (2021) [24]	18,405	SSZ	10,789	52, 260, 520	65	68.7	412	N/A	N/A	Register-based diagnostic codes	General ILD, drug-induced ILD	Moderate	Nationwide, population-based, retrospective cohort study
Juge et al. (2021) [25]	929	N/A	N/A	312. 676	63	59	307	58.2	83.3	HRCT	UIP	Moderate	Multicenter, retrospective case-control study
Li et al. (2020) [26]	316	LEF, tripterygium, HCQ, SSZ, TNFi	208	221	52.62	72.59	83	106	230	HRCT	UIP, NSIP, OP, LIP	Moderate	Retrospective cohort study
Robles-Pérez et al. (2020) [27]	50	Leflunomide, prednisone, biologics	-	260	47.1	75	6	41.7	N/A	PFT, DLCO, FVC, HRCT	NSIP, OP, UIP	Low	Single-center prospective cohort study
Kiely et al. (2019) [28]	1,578	SSZ, HCQ	1,114	1,300	62	66.9	39	19	60.7	CRF, PFT, HRCT, CXR	ILD/pulmonary fibrosis	Low	Prospective observational cohort study
Sparks et al. (2019) [29]	1,061	Non-biologic DMARDS, biologic DMARDS, glucocorticoids	1,336, 663, 1,129	463	55.8	82.3	85	N/A	68.6	Chest CT, PFTs	NSIP, UIP	Moderate	Prospective cohort study
Curtis et al. (2015) [30]	7,043	Anti-TNF, tocilizumab, rituximab, abatacept	13,296	36	52	82	16	N/A	N/A	ICD-9 coding, CT	Pulmonary fibrosis, IIP	Serious	Retrospective cohort study
Kur-Zalewska et al. (2021) [31]	86	Sulfasalazine, cyclosporine, gold salts, chloroquine, leflunomide, biologics	N/A	112	60.5	85.32	74	53	77.57	HRCT	RA-ILD	Low	Observational, cross-sectional study
Izuka et al. (2021) [32]	99	Non-MTX	66	676	73.6	71.9	165	97.85	89.3	HRCT	UIP, ILD-AE, non-UIP	Low	Retrospective observational study
Provan et al. (2024) [33]	16, 330	N/A	N/A	260	55.6	74.86	225	6,584	80.78	ICD-10 code	Other interstitial pulmonary disease	Moderate	Multicenter, observational cohort study

Discussion

Methotrexate is one of the leading prescribed medications for the long-term management of patients with RA. As emphasized by the American College of Rheumatology (ACR) in its 2021 guideline, methotrexate is considered the anchor drug and first-line disease-modifying anti-rheumatic drug (DMARD) for most patients with RA [35]. Concerns about methotrexate-induced pulmonary toxicity have persisted for decades [13,14]. These concerns stem primarily from early reports of methotrexate-associated lung injury and ongoing debates regarding its potential role in exacerbating or precipitating ILD in patients with RA. However, as new evidence emerges, the 2023 American College of Rheumatology, 2025 European Respiratory Society (ERS), and European League Against Rheumatism (EULAR) guidelines do not recommend methotrexate as first-line therapy for ILD. While methotrexate has historically been controversial due to concerns about pulmonary toxicity, recent evidence indicates it is not associated with increased risk of ILD development or progression, particularly in RA-ILD [35-39].

Although there is broad support for the conclusion that methotrexate does not cause RA-ILD, some studies continue to report adverse pulmonary effects. Numerous studies have shown that methotrexate and RA-ILD are significantly associated but emphasize that association does not equal causation and highlight the importance of considering confounders such as disease severity, duration, and comorbid risk factors [26-29]. These studies include the influence of confounding factors such as RA disease severity, longer disease duration, age, smoking status, and comorbidities, all of which independently increase the risk of ILD. The variability in patient populations and diagnostic criteria further limits the generalizability of these findings.

Meta-analyses of randomized controlled trials have helped distinguish between acute and chronic toxicities associated with methotrexate [40]. These studies indicate that methotrexate carries a small but genuine risk of acute hypersensitivity pneumonitis. This early reaction is clinically and pathologically distinct from the chronic fibrosing interstitial lung disease seen in RA [40-42]. This condition is rare and non-dose-dependent and typically reversible with prompt drug discontinuation. In contrast, there is no convincing evidence that methotrexate contributes to the progression of chronic ILD or noninfectious respiratory events in patients with RA. Other reviews have highlighted the unique risk of acute hypersensitivity pneumonitis while also noting the potential anti-inflammatory protective role of MTX [43,44]. Interestingly, some studies have observed a dose-dependent protective effect, in which methotrexate-treated patients exhibited lower rates and reduced severity of ILD due to methotrexate’s anti-inflammatory properties [25,31].

Although the search was comprehensive and followed PRISMA guidelines, several limitations persist in the available studies. Of the included studies, many were of moderate quality and heterogeneous, and the diagnostic criteria for ILD varied widely across cohorts [21,24-30]. Additionally, limited information on disease severity and the timing of methotrexate initiation restricted the ability to control for these confounding variables [37,38]. The lack of uniform definitions and diagnostic thresholds across studies remains a significant barrier to drawing definitive conclusions. Another potential flaw in the study’s screening process may be that individuals chosen for methotrexate treatment are already healthier, particularly in terms of pulmonary health, which introduces the possibility of selection bias. Additionally, the included studies demonstrated a degree of heterogeneity in terms of study design, population characteristics, and outcome measures. This variability likely reflects differences in institutional practices, data collection methods, and definitions used across studies. As a result, direct comparison and quantitative synthesis of findings were limited. These insights illustrate the challenges involved in drawing firm conclusions about MTX’s impact on lung health.

However, the inclusion of diverse studies provides a broader understanding of the topic and highlights consistent patterns that can be observed across varying contexts. One notable pattern observed in this review is the potential role of ethnic and geographic variation. Several studies have shown a strong positive correlation in patients of Asian descent, including those from China, Japan, and South Korea [9,45-46]. HLA subtypes, occupational exposures, and variations in diagnostic practices are common genetic and environmental differences that influence both the prevalence of RA-ILD and the pulmonary effects of methotrexate [9,45-48]. These findings highlight the need for ethnically diverse, multicenter investigations that account for genetic predisposition, cultural variations in healthcare delivery, and variable treatment practices across global populations.

The findings support the American College of Rheumatology guidelines, as many studies indicate that MTX is not significantly associated with the development of RA-ILD. However, other population-based studies have shown that the increased risk of ILD in patients with RA is attributable to the underlying disease rather than methotrexate exposure, but further research must be conducted to disentangle the effects of methotrexate from those of RA itself and other DMARDs, especially in patients with pre-existing or subclinical lung disease [34]. Further research is needed on extensive, prospective, multi-ethnic cohort studies with standardized definitions to clarify whether methotrexate causes RA-ILD.

## Conclusions

With the growing recognition of pulmonary complications in rheumatoid arthritis, questions about methotrexate’s role in interstitial lung disease remain unresolved. Although some reports suggest methotrexate may trigger or worsen RA-ILD, most studies do not support a consistent or causal link. On the contrary, several findings point toward a possible protective effect, likely through its ability to reduce systemic inflammation and limit lung involvement. Still, important gaps remain, as differences in study design, diagnostic methods, and patient characteristics, along with genetic and regional factors, continue to cloud interpretation. Large, multicenter studies with standardized definitions are needed to clarify methotrexate’s true impact. Ultimately, methotrexate may prove to be neutral, or even protective, in the complex relationship between rheumatoid arthritis and interstitial lung disease.

## References

[1] (2023). Global, regional, and national burden of rheumatoid arthritis, 1990-2020, and projections to 2050: a systematic analysis of the Global Burden of Disease Study 2021. Lancet Rheumatol.

[2] McInnes IB, Schett G (2011). The pathogenesis of rheumatoid arthritis. N Engl J Med.

[3] Johnson SR, Bernstein EJ, Bolster MB (2024). 2023 American College of Rheumatology (ACR)/American College of Chest Physicians (CHEST) guideline for the treatment of interstitial lung disease in people with systemic autoimmune rheumatic diseases. Arthritis Care Res (Hoboken).

[4] Gregersen PK, Silver J, Winchester RJ (1987). The shared epitope hypothesis. An approach to understanding the molecular genetics of susceptibility to rheumatoid arthritis. Arthritis Rheum.

[5] Darrah E, Andrade F (2018). Rheumatoid arthritis and citrullination. Curr Opin Rheumatol.

[6] Alghamdi M, Alasmari D, Assiri A, Mattar E, Aljaddawi AA, Alattas SG, Redwan EM (2019). An overview of the intrinsic role of citrullination in autoimmune disorders. J Immunol Res.

[7] Deane KD, Nicolls MR (2013). Developing better biomarkers for connective tissue disease-associated interstitial lung disease: citrullinated hsp90 autoantibodies in rheumatoid arthritis. Arthritis Rheum.

[8] Bendstrup E, Møller J, Kronborg-White S, Prior TS, Hyldgaard C (2019). Interstitial lung disease in rheumatoid arthritis remains a challenge for clinicians. J Clin Med.

[9] Kim Y, Yang HI, Kim KS (2023). Etiology and pathogenesis of rheumatoid arthritis-interstitial lung disease. Int J Mol Sci.

[10] Benjamin O, Goyal A, Lappin SL (2025). Disease-modifying antirheumatic drugs (DMARD). StatPearls.

[11] Jakubovic BD, Donovan A, Webster PM, Shear NH (2013). Methotrexate-induced pulmonary toxicity. Can Respir J.

[12] Chhabra P, Law AD, Suri V, Malhotra P, Varma S (2012). Methotrexate induced lung injury in a patient with primary CNS lymphoma: a case report. Mediterr J Hematol Infect Dis.

[13] Kadura S, Raghu G (2021). Rheumatoid arthritis-interstitial lung disease: manifestations and current concepts in pathogenesis and management. Eur Respir Rev.

[14] Peasah SK, Swart EC, Huang Y (2024). Disease-modifying medications in patients with rheumatoid arthritis in the USA: trends from 2016 to 2021. Drugs Real World Outcomes.

[15] Chang SH, Jung S, Chae JJ (2024). Therapeutic single-cell landscape: methotrexate exacerbates interstitial lung disease by compromising the stemness of alveolar epithelial cells under systemic inflammation. EBioMedicine.

[16] Dinache G, Popescu CC, Mogoșan C, Enache L, Agache M, Codreanu C (2022). Lung damage in rheumatoid arthritis-a retrospective study. Int J Mol Sci.

[17] Huang S, Doyle TJ, Hammer MM (2020). Rheumatoid arthritis-related lung disease detected on clinical chest computed tomography imaging: prevalence, risk factors, and impact on mortality. Semin Arthritis Rheum.

[18] Hozumi H, Nakamura Y, Johkoh T (2013). Acute exacerbation in rheumatoid arthritis-associated interstitial lung disease: a retrospective case control study. BMJ Open.

[19] Zhou W, Yang Y, Su L (2025). Development and validation of predictive factors influencing rheumatoid arthritis associated with interstitial lung disease. Clin Rheumatol.

[20] Page MJ, Moher D, Bossuyt PM (2021). PRISMA 2020 explanation and elaboration: updated guidance and exemplars for reporting systematic reviews. BMJ.

[21] Chamizo-Carmona E, Carrasco Cubero C, Rojas Herrera SM, Malave Calzada J, Veroz Gonzalez R, Chaves Chaparro LM (2018). Interstitial lung disease in patients with rheumatoid arthritis of a cohort treated with methotrexate monotherapy. Ann Rheum Dis.

[22] Genc AC, Ozturk Z, Kara AB, Turkoglu Genc F, Aydemir Y, Gunduz Y, Gonullu E (2023). Assessment of the clinical features of rheumatoid arthritis-related interstitial lung diseases: a retrospective evaluation. Eur Rev Med Pharmacol Sci.

[23] Severo CR, Chomiski C, Valle MB, Escuissato DL, Paiva ED, Storrer KM (2022). Assessment of risk factors in patients with rheumatoid arthritis-associated interstitial lung disease. J Bras Pneumol.

[24] Kim K, Woo A, Park Y (2022). Protective effect of methotrexate on lung function and mortality in rheumatoid arthritis-related interstitial lung disease: a retrospective cohort study. Ther Adv Respir Dis.

[25] Ibfelt EH, Jacobsen RK, Kopp TI (2021). Methotrexate and risk of interstitial lung disease and respiratory failure in rheumatoid arthritis: a nationwide population-based study. Rheumatology (Oxford).

[26] Juge PA, Lee JS, Lau J (2021). Methotrexate and rheumatoid arthritis associated interstitial lung disease. Eur Respir J.

[27] Li L, Liu R, Zhang Y (2020). A retrospective study on the predictive implications of clinical characteristics and therapeutic management in patients with rheumatoid arthritis-associated interstitial lung disease. Clin Rheumatol.

[28] Robles-Pérez A, Luburich P, Bolivar S, Dorca J, Nolla JM, Molina-Molina M, Narváez J (2020). A prospective study of lung disease in a cohort of early rheumatoid arthritis patients. Sci Rep.

[29] Kiely P, Busby AD, Nikiphorou E (2019). Is incident rheumatoid arthritis interstitial lung disease associated with methotrexate treatment? Results from a multivariate analysis in the ERAS and ERAN inception cohorts. BMJ Open.

[30] Sparks JA, He X, Huang J (2019). Rheumatoid arthritis disease activity predicting incident clinically apparent rheumatoid arthritis-associated interstitial lung disease: a prospective cohort study. Arthritis Rheumatol.

[31] Curtis JR, Sarsour K, Napalkov P, Costa LA, Schulman KL (2015). Incidence and complications of interstitial lung disease in users of tocilizumab, rituximab, abatacept and anti-tumor necrosis factor α agents, a retrospective cohort study. Arthritis Res Ther.

[32] Kur-Zalewska J, Kisiel B, Kania-Pudło M, Tłustochowicz M, Chciałowski A, Tłustochowicz W (2021). A dose-dependent beneficial effect of methotrexate on the risk of interstitial lung disease in rheumatoid arthritis patients. PLoS One.

[33] Izuka S, Yamashita H, Iba A, Takahashi Y, Kaneko H (2021). Acute exacerbation of rheumatoid arthritis-associated interstitial lung disease: clinical features and prognosis. Rheumatology (Oxford).

[34] Provan SA, Ljung L, Kristianslund EK (2024). Interstitial lung disease in patients with rheumatoid arthritis or psoriatic arthritis initiating biologics and controls: data from 5 Nordic registries. J Rheumatol.

[35] Fraenkel L, Bathon JM, England BR (2021). 2021 American College of Rheumatology guideline for the treatment of rheumatoid arthritis. Arthritis Care Res (Hoboken).

[36] Aletaha D, Smolen JS (2018). Diagnosis and management of rheumatoid arthritis: a review. JAMA.

[37] Johnson SR, Bernstein EJ, Bolster MB (2024). 2023 American College of Rheumatology (ACR)/American College of Chest Physicians (CHEST) guideline for the treatment of interstitial lung disease in people with systemic autoimmune rheumatic diseases. Arthritis Rheumatol.

[38] Antoniou KM, Distler O, Gheorghiu AM (2025). ERS/EULAR clinical practice guidelines for connective tissue disease-associated interstitial lung diseaseDeveloped by the task force for connective tissue disease-associated interstitial lung disease of the European Respiratory Society (ERS) and the European Alliance of Associations for Rheumatology (EULAR)Endorsed by the European Reference Network on rare respiratory diseases (ERN-LUNG). Eur Respir J.

[39] Mahler B, Moșteanu MI, Bobocea R (2024). Multiple pulmonary involvement in the rapidly progressive evolution of rheumatoid arthritis. Diagnostics (Basel).

[40] Conway R, Low C, Coughlan RJ, O'Donnell MJ, Carey JJ (2014). Methotrexate and lung disease in rheumatoid arthritis: a meta-analysis of randomized controlled trials. Arthritis Rheumatol.

[41] Fragoulis GE, Conway R, Nikiphorou E (2019). Methotrexate and interstitial lung disease: controversies and questions. A narrative review of the literature. Rheumatology (Oxford).

[42] Fragoulis GE, Nikiphorou E, Larsen J, Korsten P, Conway R (2019). Methotrexate-associated pneumonitis and rheumatoid arthritis-interstitial lung disease: current concepts for the diagnosis and treatment. Front Med (Lausanne).

[43] Fleischmann R, Mysler E, Hall S (2017). Efficacy and safety of tofacitinib monotherapy, tofacitinib with methotrexate, and adalimumab with methotrexate in patients with rheumatoid arthritis (ORAL Strategy): a phase 3b/4, double-blind, head-to-head, randomised controlled trial. Lancet.

[44] Fleischmann R, Schiff M, van der Heijde D (2017). Baricitinib, methotrexate, or combination in patients with rheumatoid arthritis and no or limited prior disease-modifying antirheumatic drug treatment. Arthritis Rheumatol.

[45] Zhang M, Yin J, Zhang X (2023). Factors associated with interstitial lung disease in patients with rheumatoid arthritis: a systematic review and meta-analysis. PLoS One.

[46] Kim H, Cho SK, Song YJ (2023). Clinical characteristics of rheumatoid arthritis patients with interstitial lung disease: baseline data of a single-center prospective cohort. Arthritis Res Ther.

[47] Dai Y, Wang W, Yu Y, Hu S (2021). Rheumatoid arthritis-associated interstitial lung disease: an overview of epidemiology, pathogenesis and management. Clin Rheumatol.

[48] Gravallese EM, Firestein GS (2023). Rheumatoid arthritis - common origins, divergent mechanisms. N Engl J Med.

